# Active transcription in the vascular bed characterizes rapid progression in idiopathic pulmonary fibrosis

**DOI:** 10.1172/JCI165976

**Published:** 2023-08-15

**Authors:** Nirmal S. Sharma, Kapil Patel, Ezgi Sari, Shruti Shankar, Maria G. Gastanadui, Diego Moncada-Giraldo, Yixel Soto-Vazquez, Delores Stacks, Louise Hecker, Kevin Dsouza, Mudassir Banday, Edward O’Neill, Paul Benson, Gregory Payne, Camilla Margaroli, Amit Gaggar

**Affiliations:** 1 Department of Medicine, Brigham and Women’s Hospital, Boston, Massachusetts, USA.; 2 West Roxbury VA Medical Center, Boston, Massachusetts, USA.; 3 Department of Medicine, University of South Florida, Tampa, Florida, USA.; 4 Department of Medicine, University of Alabama at Birmingham, Birmingham, Alabama, USA.; 5 Department of Pediatrics, Emory University, Atlanta, Georgia, USA.; 6 Department of Pathology, University of Alabama at Birmingham, Birmingham, Alabama, USA.; 7 Department of Medicine, Emory University, Atlanta, Georgia, USA.; 8 Birmingham VA Medical Center, Birmingham, Alabama, USA.; 9 Lung Health Center, Program in Protease and Matrix Biology, and Gregory Fleming James CF Center, University of Alabama at Birmingham, Birmingham, Alabama, USA.

**Keywords:** Pulmonology, Vascular Biology, Fibrosis

**To the Editor:** Idiopathic pulmonary fibrosis (IPF) is the most common manifestation of interstitial lung disease, with a median survival of 3–5 years after diagnosis. IPF is characterized by progressive fibrosis with the development of fibroblastic foci in the interstitium. Despite the short median survival, there is a striking variance in the clinical course of IPF, with some patients characterized as stable progressors (SP) and some as rapid progressors (RP) based on their decline in lung function and loss of forced vital capacity (FVC) (1). While the presence of these two patient groups is well known, the biological mechanisms defining this dichotomy are poorly understood.

To investigate these differences, we collected lung biopsies from patients with IPF at the time of diagnosis and followed their disease progression (IRB PRO0032158, Supplemental Table 1; supplemental material available online with this article; https://doi.org/10.1172/JCI165976DS1). We identified 5 SP and 4 RP at the time of diagnosis based on loss of at least 10% FVC within the first year as well as 3 patients with terminal-stage IPF delineated for transplant (TP) and 3 individuals with age-matched normal lung tissues (NC) (clear margins from excision for pulmonary hamartoma). We investigated the presence of key transcriptional signatures using spatial transcriptomics (2). Briefly, sequential cuts of lung biopsies were stained with Trichrome or H&E and immunofluorescently labeled to identify nuclei, fibroblasts (α smooth muscle actin [αSMA]), and the vascular bed (CD31). Using a combination of H&E and immunofluorescent staining, regions of interest (ROIs) were selected for transcriptional analysis (see Supplemental Methods).

First, we sought to investigate whether transcriptional signatures of ROIs showing key features of IPF (collagen deposition and presence of fibroblastic foci) were sufficient to discriminate between SP and RP (Figure 1, A and B). SP samples showed increased expression of immunoglobulin genes (Figure 1C), while RP samples had increased expression of surfactant proteins A1, A2, and C, for which genetic variants have been associated with poor prognosis in patients with IPF (3). Furthermore, *CCN1*, a protein expressed in fibroblasts at homeostasis and highly induced in dysregulated fibrosis (4), was found to be significantly increased in RP.

While these differences were present in the overall transcriptome, they were not fully reflected in the specific cell populations (Supplemental Figure 1A), prompting a closer analysis of the different cell types. Unlike the general areas and the fibroblastic foci (Supplemental Figure 1, B–D), the vasculature in the RP samples (Figure 1D) was more transcriptionally active, with an increased proportion of differentially expressed genes compared with SP and TP samples (Figure 1, E–G, and Supplemental Figure 1, E–G), but similarly active compared with NC samples (73 upregulated genes in NC and 70 in RP), suggesting a potentially novel avenue to investigate the role of pulmonary vasculature in the progression of IPF. At the transcriptional level, upregulated genes in the RP vascular bed, compared with that of SP, TP, and NC, including *LRRC38*, *LUM*, *COA5*, and *ITM2A,* were mainly under the control of TBX3 (Supplemental Figure 1H), a member of a transcription factor family overexpressed in models of lung fibrosis (5) (Figure 1H and Supplemental Figure 1I). These differences were also corroborated by comparison with publicly available single-cell RNA-Seq data sets that included endothelial cells (http://www.ipfcellatlas.com/) (Supplemental Table 2), and TBX3 expression at the protein level was confirmed by immunofluorescence (Supplemental Figure 1J).

Given the increased transcriptional activity found in the vasculature of the RP group, we investigated whether there could be transcriptional crosstalk between the vascular bed and the fibroblastic foci. In the fibroblastic foci (Supplemental Figure 1D), distinct *HLA-DQ* haplotypes were expressed in the SP and RP groups, suggesting differential immune responses in the two groups. Furthermore, *MMP7* was highly expressed in the RP group compared with that in the SP and TP groups (Supplemental Figure 1, D and I), strengthening its role as a biomarker for rapid FVC decline. The role of MMP7 in the reorganization of the extracellular matrix is well known. However, MMP7 can serve as a crosstalk mediator between the secreting cells and the endothelium. Indeed, MMP7 indirectly promotes angiogenesis via the cleavage of the soluble VEGF receptor 1, and the cleavage of CCN2, resulting in the increased bioavailability of VEGF and reactivation of its angiogenic activity (6). While our data suggest a crosstalk between fibroblasts and the vascular bed, future studies will be needed to better understand other targets and interactions between cell types involved in lung fibrosis, including the effect of VEGF in the interaction among the capillary endothelial cells, the epithelium, and the inflammatory cells.

In conclusion, this study suggests a key role of the vascular bed in the progression of IPF, warranting further investigations. This study has limitations owing to its preliminary nature; use of a large control group with minimal lung disease would allow for statistically robust multivariate analysis in future larger studies. Furthermore, these results show similarities with publicly available single-cell transcriptional data sets, and they also highlight key differences. These could be attributed either to technical aspects of transcriptional profiling or selection of specific IPF subphenotypes. Future studies with a controlled patient cohort will be needed to discern the true variability between gene profiling by single-cell RNA-Seq and spatial transcriptomics. Nevertheless, these data highlight distinct pathways of crosstalk between fibroblasts and the vascular bed, which could prove central to the pathophysiology of the disease. Targeting such pathways and modulating the interaction between the vasculature and fibroblasts may usher the development of future therapeutics to limit the progression of IPF.

## Supplementary Material

Supplemental data

Supporting data values

## Figures and Tables

**Figure 1:**
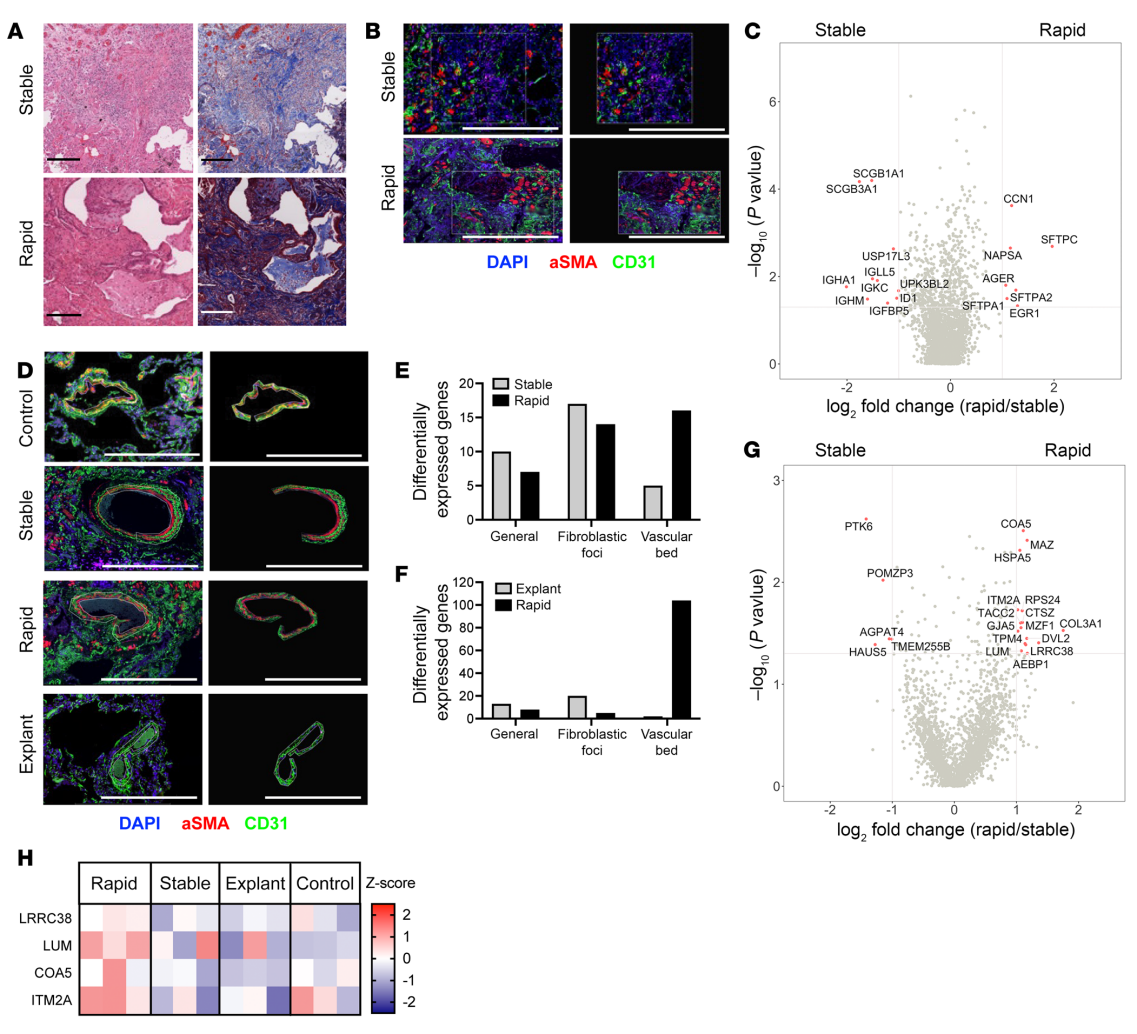
Transcriptional profile of the IPF lung shows unique signatures. (**A**) Lung biopsies from patients with IPF characterized as SP or RP stained with H&E (left) or Trichrome (right). Scale bar: 250 μm. (**B**) Immunofluorescence staining and ROI selection. Scale bar: 500 μm. (**C**) Differential gene expression analysis (SP = 5 patients; RP = 4 patients). (**D**) Immunofluorescence staining and ROI selection for vascular bed. Scale bar: 500 μm. (**E** and **F**) Number of upregulated genes. (**G**) Differential gene expression analysis of vascular bed. (**H**) Unique upregulated genes in the RP vascular bed. Significance for differential gene expression: log_2_ fold change of 1 or –1, *P* = 0.05.

## Version 1 06/29/2023

In-Press Preview

## Version 2 08/15/2023

Electronic publication
