# Supplementary material for: Active transcription in the vascular bed characterizes rapid progression in idiopathic pulmonary fibrosis
Source: J Clin Invest. 2023 Aug 15;133(16):e165976. doi: 10.1172/JCI165976 (PMC10425209; doi:10.1172/JCI165976)
Supplement: Supplemental data [file jci-133-165976-s062.pdf]

Supplement for

Active transcription in the vascular bed characterizes rapid progression in idiopathic pulmonary fibrosis

Nirmal S. Sharma[1,2*], Kapil Patel[3*], Ezgi Sari[4], Shruti Shankar[3], Maria G. Gastanadui[4], Diego Moncada-Giraldo[5], Yixel Soto-Vazquez[4], Delores Stacks[6], Louise Hecker[7], Kevin Dsouza[4], Mudassir Banday[1], Edward O'neill[3], Paul Benson[6], Gregory Payne[4,8,9], Camilla Margaroli[4,6,8#], Amit Gaggar[4,8,9#]

Supplemental methods

Figures: 1

Tables: 2

Supplemental methods

Spatial transcriptomics

Paraffin-embedded 5μm tissue cuts were baked at 60°C for 1 hour and were processed following manufacturer's protocol (2). RNA was profiled using the human WTA probes (Nanostring Technologies) and cells of interest were stained for nuclei (Syto61, Thermofisher), for α-Smooth muscle actin (Abcam, ab202368, clone 1A4), and for CD31 (Abcam, ab9498, clone JC/70A). Regions of interest (ROIs) were selected by two trained pathologists using a combination of immunofluorescent staining, Trichrome staining, H&E staining. ROIs were classified as general areas showing key features of IPF (mixed cell composition in areas showing collagen deposition and presence of fibroblastic foci), as vascular beds, or as fibroblastic foci. Upon ROI collection on the GeoMx Digital Spatial Profiler (DSP) (Nanostring Technologies), libraries were prepared following manufacturer's protocol (Nanostring Technologies) and sequenced on the Illumina NovaSeq instrument (MedGenome Inc). FASTQ files were uploaded on the Basespace Illumina hub and converted to digital count conversion (.dcc) files using the GeoMx® NGS Pipeline (v2.0.21) on Illumina DRAGEN. DCC files were uploaded and analyzed on the GeoMx DSP Analysis suite (v2.4.0.421). ROIs with less than 80% sequencing alignment, or less than 50% sequencing saturation were removed from the analysis. Outlier probes were identified by Grubbs outlier test. Limit of quantification (LOQ) was calculated as 2 standard deviations above the geometric mean of the negative probes. ROIs were then divided as general areas, vascular bed, and fibroblastic foci and kept separated by tissue type for the remainder of the analysis. Genes were removed from the analysis if their counts were found to be below the LOQ in at least 10% of the ROIs. Then counts were normalized using a signal-based quartile normalization, in which individual counts are normalized against the 75th percentile of signal from their own ROI. Normalized counts were used for statistical analysis using a mixed linear model as previously described (2). Differential

gene expression was considered significant with a log2 fold change higher than 1 and a p-value cutoff of 0.05. Normalized counts have been can be accessed here: 10.17632/9mckhpcdjw.1 . Protein expression of selected genes was validated by immunofluorescent staining for MMP-7 (Abcam, ab232737), Tbx3 (Abcam, ab99302), COA5 (Thermofisher, PA5-63480).

Table S1

Patient status	Age	Gender	Race	Baseline FVC	Baseline FVC (%)	Baseline TLC (L)	Baseline TLC (%)
Stable	66	M	H	2.07	67	2.6	43
Stable	67	F	C	1.63	64	2.84	66
Stable	60	M	C	1.86	42	2.87	43
Stable	59	M	C	2.48	63	3.63	62
Stable	53	M	C	1.79	42	3.04	49
Rapid	54	F	A	2.33	78	2.96	65
Rapid	68	M	C	2.24	49	3.61	52
Rapid	60	F	C	2.29	84	3.42	74
Rapid	63	M	C	2.98	73	4.69	74
Explant	59	M	C	1.75	37	2.59	37
Explant	67	F	AA	2.72	76	4.12	71
Explant	65	M	C	1.54	33	2.65	47
Control	77	M	C				
Control	69	F	AA				
Control	72	F	H				

Table S1. Summary of patient demographics. Abbreviations: TLC = Total Lung Capacity; FVC = Forced Vital Capacity; M = male; F = female; A = Asian; AA = African American; C = Caucasian; H = Hispanic.

Gene expression in vasculature	Current study	Kaminiski Database	Banovich Database
TBX3	Increased expression in rapid vs stable IPF	Increased expression in IPF vs healthy controls	Increased expression in IPF vs healthy controls
LRRC38	Increased expression in rapid vs stable IPF	Expressed in IPF endothelial cells	No increase seen in endothelial cells
LUM	Increased expression in rapid vs stable IPF	No increased expression in endothelial	No increased expression in endothelial
ITM2A	Increased expression in rapid vs stable IPF	Increased expression in IPF vs healthy controls	Increased expression in IPF vs healthy controls
DEPP1	Trend towards increase in rapid compared to stable IPF	Increased expression in IPF vs healthy controls	No increased expression in IPF vs healthy controls
COA5	Increased expression in rapid vs stable IPF	Increased expression in IPF vs healthy controls	Increased expression in IPF vs healthy controls
COL3A1	Increased expression in rapid vs stable IPF	No increased expression in IPF vs healthy controls	No increased expression in IPF vs healthy controls

Table S2. Comparison of vascular gene expression with public scRNA-seq data.

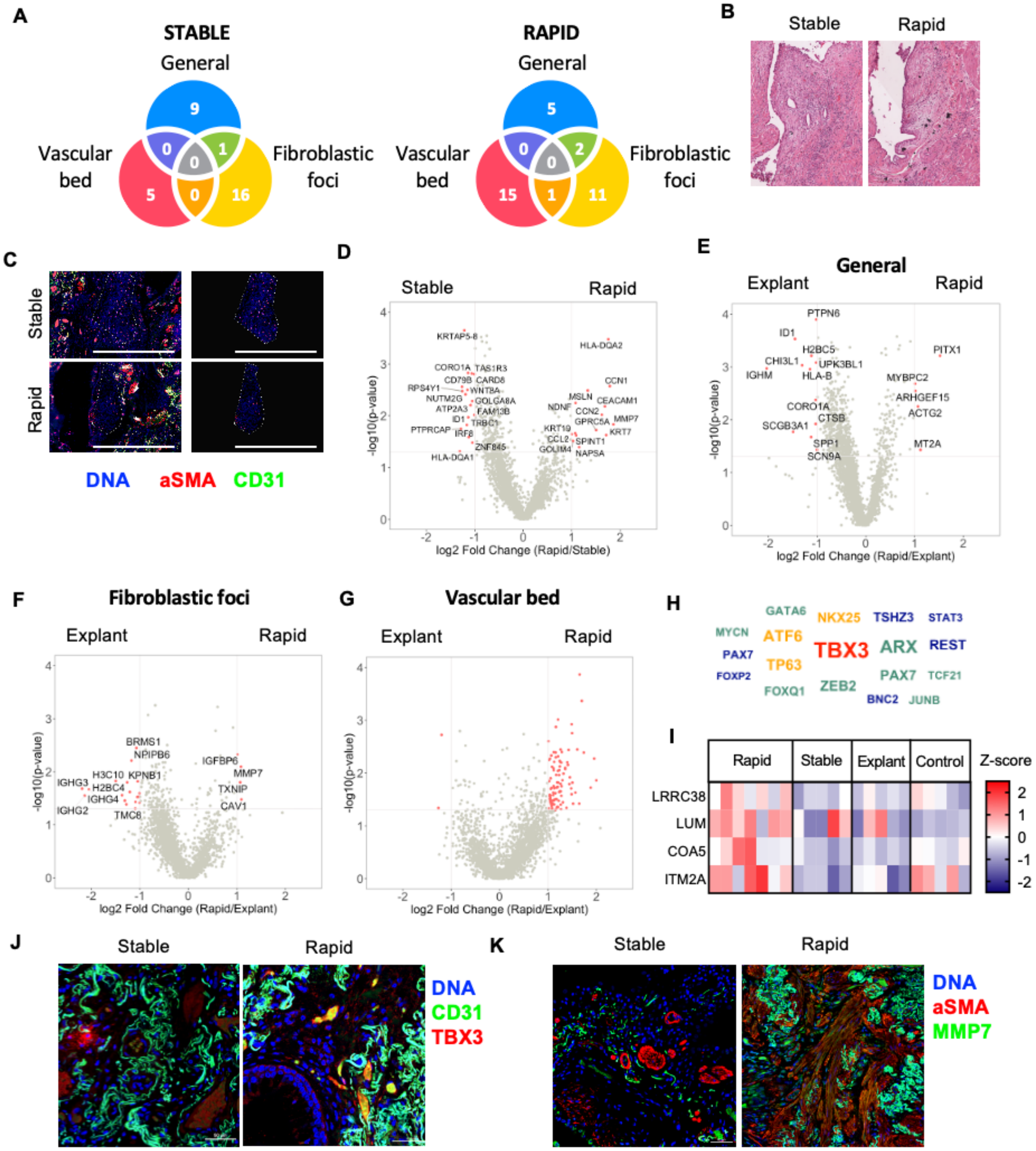

Fig. S1. Rapid show unique transcriptional profile. (A) Comparison of differentially expressed genes in ROIs of general areas, fibroblastic foci, and vasculature for stable patients (left) and rapid progressors (right) **(B)** Lung biopsies from IPF patients stained by H&E focused on fibroblastic foci (scale bar = 250μm). **(C)** Immunofluorescence staining and ROI selection for fibroblastic foci (scale bar = 500μm). **(D)** Differential gene expression analysis of fibroblastic

foci. **(E-G)** Differential gene expression analysis of rapid progressors and explanted IPF lungs. **(H)** Analysis of transcription factors regulating differentially expressed genes in the vasculature of rapid progressors. (Size = number of genes regulated; Red-Yellow-Green-Blue = lower to higher p-value) **(I)** Unique upregulated genes in the rapid progressor vascular bed. Each column represents one ROI. **(J)** Immunofluorescent staining of stable and rapid lung tissue for TBX3 (red), CD31 (green), nuclei (blue). **(K)** MMP7 expression in stable and rapid lung tissue (MMP7 green, aSMA red, nuclei blue). Significance for differential gene expression: log2 fold change 1 or -1, p-value = 0.05.
